# Extreme genetic fragility of the HIV-1 capsid

**DOI:** 10.1186/1742-4690-10-S1-P73

**Published:** 2013-09-19

**Authors:** Suzannah Rihn, Sam Wilson, Nick Loman, Mudathir Alim, Saskia Bakker, David Bhella, Frazer Rixon, Paul Bieniasz

**Affiliations:** 1 Laboratory of Retrovirology, The Rockefeller University, New York, NY, USA; 2 Aaron Diamond AIDS Research Center, The Rockefeller University, New York, NY, USA; 3 Howard Hughes Medical Institute, Aaron Diamond AIDS Research Center, New York, NY, USA; 4 Centre for Systems Biology, University of Birmingham, Birmingham, UK; 5 MRC Centre for Virus Research, Institute of Infection, Immunity and Inflammation, College of Medical, Veterinary and Life Sciences, University of Glasgow, Glasgow, UK

## 

Genetic robustness, or fragility, is defined as the ability, or lack thereof, of a biological entity to maintain function in the face of mutations. Viruses that replicate via RNA intermediates exhibit high mutation rates, and robustness should be particularly advantageous to them. The capsid (CA) domain of the HIV-1 Gag protein is under strong pressure to conserve functional roles in viral assembly, maturation, uncoating, and nuclear import. However, CA is also under strong immunological pressure to diversify in hosts. Therefore, it would be particularly advantageous for CA to evolve genetic robustness. To probe the genetic robustness of HIV-1 CA, we generated a library of single amino acid substitution mutants, encompassing almost half the residues in CA. Strikingly, we found HIV-1 CA to be the most genetically fragile protein that has been analyzed using such an approach, with 70% of mutations yielding replication-defective viruses. Although CA participates in several steps in HIV-1 replication, analysis of conditionally (temperature sensitive) and constitutively non-viable mutants revealed that the biological basis for its genetic fragility was primarily the need to coordinate the efficient and proper assembly of a mature viral capsid. All mutations that exist in naturally occurring HIV-1 subtype B populations at a frequency >3%, and were also present in the mutant library, had fitness levels that were >40% of WT. However, some mutations with high fitness did not occur in natural populations, suggesting another form of selection pressure limiting variation *in vivo.* Additionally, known protective CTL epitopes occurred preferentially in domains of the HIV-1 CA that were even more genetically fragile than HIV-1 CA as a whole. The extreme genetic fragility of HIV-1 CA may be one reason why cell-mediated immune responses to Gag correlate with better prognosis in HIV-1 infection, and suggests that CA is a good target for therapy and vaccination strategies.

